# A hybridized red deer and rough set clinical information retrieval system for hepatitis B diagnosis

**DOI:** 10.1038/s41598-024-53170-5

**Published:** 2024-02-15

**Authors:** Madhusmita Mishra, D. P. Acharjya

**Affiliations:** Vellore Institute of Technology, School of Computer Science and Engineering, Vellore, 632014 India

**Keywords:** Computational biology and bioinformatics, Health care

## Abstract

Healthcare is a big concern in the current booming population. Many approaches for improving health are imposed, such as early disease identification, treatment, and prevention. Therefore, knowledge acquisition is highly essential at different stages of decision-making. Inferring knowledge from the information system, which necessitates multiple steps for extracting useful information, is one technique to address this problem. Handling uncertainty throughout data analysis is also another challenging task. Computer intelligence is a step forward to this end while selecting characteristics, classification, clustering, and developing clinical information retrieval systems. According to recent studies, swarm optimization is a useful technique for discovering key features while resolving real-world issues. However, it is ineffective in managing uncertainty. Conversely, a rough set helps a decision system generate decision rules. This produces decision rules without any additional information. In order to assess real-world information systems while managing uncertainties, a hybrid strategy that combines a rough set and red deer algorithm is presented in this research. In the red deer optimization algorithm, the suggested method selects the optimal characteristics in terms of the degree of dependence on the rough set. In order to determine the decision rules, further a rough set is used. The efficiency of the suggested model is also contrasted with that of the decision tree algorithm and the conventional rough set. An empirical study on hepatitis disease illustrates the viability of the proposed research as compared to the decision tree and crisp rough set. The proposed hybridization of rough set and red deer algorithm achieves an accuracy of 91.7% accuracy. The acquired accuracy for the decision tree, and rough set methods is 82.9%, and 88.9%, respectively. It suggests that the proposed research is viable.

## Introduction

Progressively data generation and its use have been increased in various sectors because of the wide spread of information and communication technology. The healthcare sector is one among many sectors which produce data exponentially. These sectors employ technologies that compress enormous volumes of data per microsecond. As data volumes increase, data analysis techniques including feature extraction, rule generation, and data reduction become more important^1^. As a basis, many knowledge inference techniques were developed and computational intelligence is widely used to this extent. Dealing with ambiguity and imprecision in decision-making processes is computational intelligence’s core objective. Knowledge gained for information systems ought to be precise, intelligible, transparent, and visually conveyed. Machine learning is frequently used for the selection of characteristics, creation of rules, categorization, and grouping of them. Healthcare applications must retain the importance of data pertaining to a certain condition by selecting features and creating decision rules. Both selections of features and production of rules have been added based on the healthcare applications.

Besides, knowledge inference’s primary issue is choosing important attributes. Recent times have seen the use of swarm algorithms to select key features to classify a system. These techniques produce outputs that are more approximate than accurate. In the literature, a variety of swarm algorithm techniques are discussed. Engineering applications employ a variety of methods, such as Particle Swarm Optimization (PSO)^2^, fish algorithm^3^, bat-inspired algorithm^4^, and whale optimization algorithm^5^ for feature selection. However, the use of these techniques in healthcare applications is limited and not widespread.

This research effort in consideration the Red Deer (RD) optimization technique^6^. The RD algorithm is meta-heuristic and imitates the natural behavior of RD. It was originally presented in 2018 and has demonstrated promising outcomes in resolving a variety of optimization issues. RD optimization is a population-based algorithm that uses a herd of RD’s collective intellect to discover the best answer to a problem. However, it deviates from PSO in some ways, including how the population moves and how the leader is chosen. Moreover, it also incorporates a few PSO characteristics, including the steering of search spaces, velocity control to control population movement, and the application of a fitness function to assess the caliber of potential solutions^7^.

For knowledge inference, on the other hand, cutting-edge computer techniques are being developed. To begin with, a fuzzy set^8^ is presented to deal with uncertainty. For knowledge inference, while controlling uncertainties, soft set^9^, Rough Set (RS)^10^, and various concepts are also introduced. Despite these equivalent procedures, RS is utilized in a variety of technical and scientific domains^11^. The variation of RS and its applications are found in emerging areas such as science and engineering^12,13^. An equivalency relation is the prime concept of RS. Further, binary relations, fuzzy equivalence relations, and intuitionistic fuzzy equivalence relations have been presented as alternatives to the equivalence relation^14,15^. clinical information retrieval systems and knowledge inference both make use of these improvements^16,17^. The RS has been expanded with numerous concepts at the same time. As an illustration, the RS has been combined with several algorithms, such as the neural network^18,19^, genetic algorithm^20^, fish swarm, cuckoo search, and shuffling frog leaping algorithm^21^. Even yet, the literature shows that the fusion of RS with swarm optimization is quite rare^22,23^. These facts show that knowledge inference frequently employs the RS, whereas a bio-inspired method is efficient for optimal feature selection.

This literature review motivated us to combine RS and the RD technique for the selection of attributes. In this study, the RS degree of dependency has been paired with the RD optimization algorithm for feature selection and knowledge discovery. While the RS effectively manages the uncertainty present in the decision system and generates the decision rules, the proposed Rough Set Red Deer (RSRD) procedure will help in finding the prime features. The main purpose of hybridizing RS with RD is that RD illustrates three key ideas of swarm intelligence techniques, such as the steering of search spaces, velocity control to control population movement, and the application of a fitness function to achieve potential solutions. The main source of inspiration for choosing the key components of this algorithm is the social behavior of red deer, which live in herds and work together to find food and avoid predators.

The article is structured as it is presented. The literature review is presented next to the introduction. The materials and methods used in this research work are presented next to the literature review. It includes the foundations of the rough set and red deer algorithm. Further, the research’s intended design is presented. Furthermore, it presents in-depth on the hepatitis B disease’s exploratory studies. It further provides an outcome analysis of the proposed research. Then, a comparative analysis is provided. Before deriving a conclusion, research contributions and limitations of the research is presented. Finally, the paper puts an end to it by bringing it to a conclusion.

## Literature review

Health informatics is the design and development of software that keeps patient information where it can be accessed by people providing care for the patient. It combines the science of healthcare with information technology. As a result, better healthcare is provided by clinicians. The clinical information retrieval system generally deals with classification, feature selection, and decision rule generation. The involved disciplines integrate the fields of computing and medicine.

Recently, classification of images are extensively carried out using convolutional neural networks^24^, deep learning^25^, and federated auto-encoding approach^26^. But, clinical information retrieval system that has been taken into consideration in this research study is not relating to images. The research survey states that Naive Bayes, artificial neural networks, and decision trees are the initial feature selection strategies used^27^. The Naive Bayes classifier using gain ratio, the decision tree using information gain, and the multi-level perceptron utilizing Chi-square are found to produce great results for feature selection. An introduction to knowledge discovery in databases using feature selection is provided by a recent survey^28^. The wrapper, filter, and embedding methods are the various feature selection techniques examined in this survey. The literature provides a review of feature selection that includes assessment metrics like correlation, mutual information, symmetric uncertainty, Euclidean distance, Laplacian score, Fisher score, and dependency index^29^.

For feature selection, a rough set-based reduct is employed in many research^17^. Without reducing the classification power of the information system, it eliminates superfluous features. It has been used to get rid of redundant features in many kinds of information systems. The biggest benefit is that data dependencies can be found without the need for additional knowledge. A thorough analysis of feature selection methods based on rough sets is available in the literature. Similar to that, a statistical solution using rough sets is provided for pattern identification and feature reduction^30^. Likewise, a backpropagation neural network and a rough set equivalence relation are utilized to create a knowledge mining model using clinical datasets^31^. In addition, a method for feature selection called feature space decomposition utilizing a rough set is created and it is analyzed over a hybrid information system^32^. The technique is protracted and hasn’t been properly evaluated on a hybridized information system, which is the main drawback. Another technique that chooses features based on boundary regions is also suggested in the literature. In this method, significant measures are constructed using the boundary region^33^.

Bio-inspired algorithms are meta-heuristic and crucial for resolving critical, time-sensitive problems. These methods become essential when there is a variation in the constraints and ignorance with limited computations. Rough and bio-inspired computing are hybridised in healthcare applications to support diagnostics, feature selection, disease categorization, and decision support. These applications are nearly invariably rife with incomplete and ambiguous data. Classification, clustering, prediction, feature selection, attribute reduction, and rule mining are challenging challenges in healthcare information system analysis. With this context, this research work hybridizes red deer optimization and RS for analyzing clinical information retrieval system.

## Materials and methods

Various methods and materials used in this research work are briefly discussed below. It includes prime notions of the RS and the foundations of the RD algorithm.

### Rudiments of rough set

The rough set is introduced to deal with uncertainties and imprecision and is recognized as an effective mathematical tool. It currently has uses in a variety of fields, such as social science, financial forecasting, fault diagnosis, and weather forecasting^34^. Here, a number of key concepts in rough set theory that are relevant to this research are discussed. Every object in the universe is defined by its features and can be represented in an information system. The objects may therefore be described using rows whereas the features are described using columns. It implies that several properties and feature values define each object.

A 4-tuple, $I=(Q, P, V, f)$, describes an information system. The notion *Q* pertains to definite objects whereas *P* relates to the features corresponding to the objects. $V=\cup _{p\in P}V_p$ confines to all feature values. A function of information is the mapping $f:(Q\times P)\rightarrow V$. If $P=(C\cup D)$, then the information system is referred to as a decision system. It is to be noted that *C* is the set of conditional attributes and *D* is the set of decisions. An equivalence relation, *IND*(*R*), as defined in Eq. (1) is the prime notion of RS where $R\subseteq (Q\times Q)$.

1 $$\begin{aligned} IND(R)=\{(q_i, q_j): f_p(q_i) = f_p(q_j)\hspace{0.2cm} \forall \hspace{0.2cm} p\in P\} \end{aligned}$$

The equivalence relation *R* divides the set *Q* into several classes which may be expressed as *Q*/*R*. Consider $X\subseteq Q$ on which the perception is to be inferred. The approximations concerning the lower and upper, represented by ${\underline{R}}X$ and ${\overline{R}}X$, respectively, approximate the *X*. Equation (2) defines the lower approximation, while Eq. (3) defines the upper approximation.

2 $$\begin{aligned} {\underline{R}}X = \cup \{Y\in Q/R: Y\subseteq X\} \end{aligned}$$

3 $$\begin{aligned} {\overline{R}}X = \cup \{Y\in Q/R: Y\cap X\ne \phi \} \end{aligned}$$

There are two situations that result from the lower and upper approximation, like ${\underline{R}}X = {\overline{R}}X$ or ${\underline{R}}X \ne {\overline{R}}X$. In the first case, *X* is a crisp set whereas *X* is a rough set in the second. The boundary line objects in the latter scenario are designated as $BN_R(X)= {\overline{R}}X - {\underline{R}}X$. Suppose there are two equivalence relations on *Q*, $A\subseteq P$, and $B\subseteq P$. The *A*-positive region of *B* is described in Eq. (4).

4 $$\begin{aligned} POS_A(B)=\cup _{X\in Q/B}\hspace{0.1cm}{\underline{A}}(X) \end{aligned}$$

The definition of $k=\gamma _A(B)$, the measure of $B's$ dependence on *A*, is elucidated in Eq. (5). *B* is independent of *A* if $k=0$. Likewise, if $k=1$, then *B* completely depends on *A*. In contrast, *B* is dependent on *A* partially and $0<k<1$.

5 $$\begin{aligned} \gamma _A(B) = k = \frac{|POS_A(B)|}{|Q|} \end{aligned}$$

The notion $\psi \rightarrow \phi$ is the common form of a decision rule. In the decision rule, conditions are denoted by the symbol $\psi$, and the decision is represented by the symbol $\phi$. Support, strength, and precision are three essential characteristics related to decision rules. The decision rule’s support is denoted by the concept $S(\psi ,\phi ) = card(||\psi \wedge \phi ||)$. Likewise, the strength of the decision rule is represented as $\sigma (\psi ,\phi ) = S(\psi ,\phi )/card(||\psi ||_{\phi })$. In Eq. (6), where $NS(\psi , \phi )$ stands for non-support of a decision rule, the accuracy of the decisions is defined.

6 $$\begin{aligned} Accy = \frac{|S(\psi , \phi )|}{|NS(\psi , \phi ) + S(\psi , \phi )|} \end{aligned}$$

A quick explanation of the rough set rule making process is given. A categorical information system is analysed as part of the rule generating algorithm to obtain candidature rules. The computational procedures involved in the rough set rule creation procedure is presented below.

**Algorithm:**
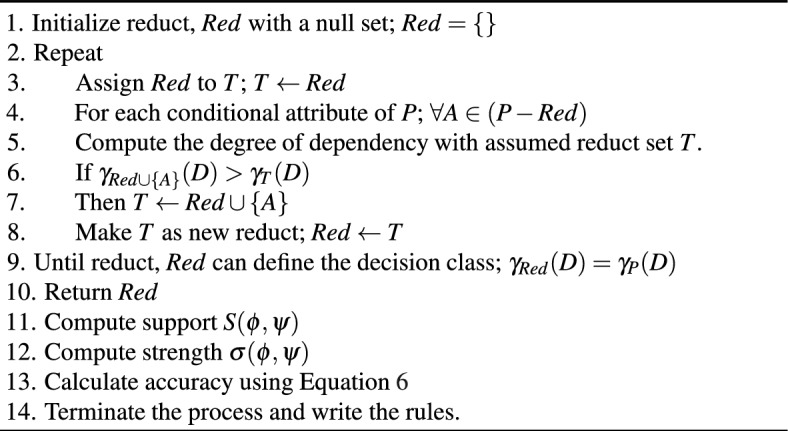
Rough set rule generation procedure.

### An analytical interpretation

Consider a liver disease diagnosis system, as indicated in Table 1. Ten patients’ worth of information is included. Five symptoms of liver disease are ascites ($p_1$), spiders ($p_2$), edema ($p_3$), bilirubin ($p_4$), and albumin ($p_5$). It shows that patient $q_1$ has ascites, spiders, no edema, very high bilirubin, and high albumin is classified as having liver disease. The remaining patients in Table 1 are likewise personified in a similar way.

**Table 1:** A sample decision table of liver disease.

Patients	$p_1$	$p_2$	$p_3$	$p_4$	$p_5$	Liver Disease ($p_d$)
$q_1$	Yes	Yes	No	Very high	High	Yes
$q_2$	No	Yes	Yes	Medium	Low	No
$q_3$	Yes	Yes	No	Very high	High	Yes
$q_4$	No	No	No	Low	Low	No
$q_5$	No	Yes	Yes	High	High	Yes
$q_6$	Yes	No	Yes	Very high	High	Yes
$q_7$	No	No	No	Low	Low	No
$q_8$	No	Yes	Yes	High	High	Yes
$q_9$	No	Yes	Yes	High	High	No
$q_{10}$	No	Yes	Yes	Medium	Low	yes

**Figure 1:**
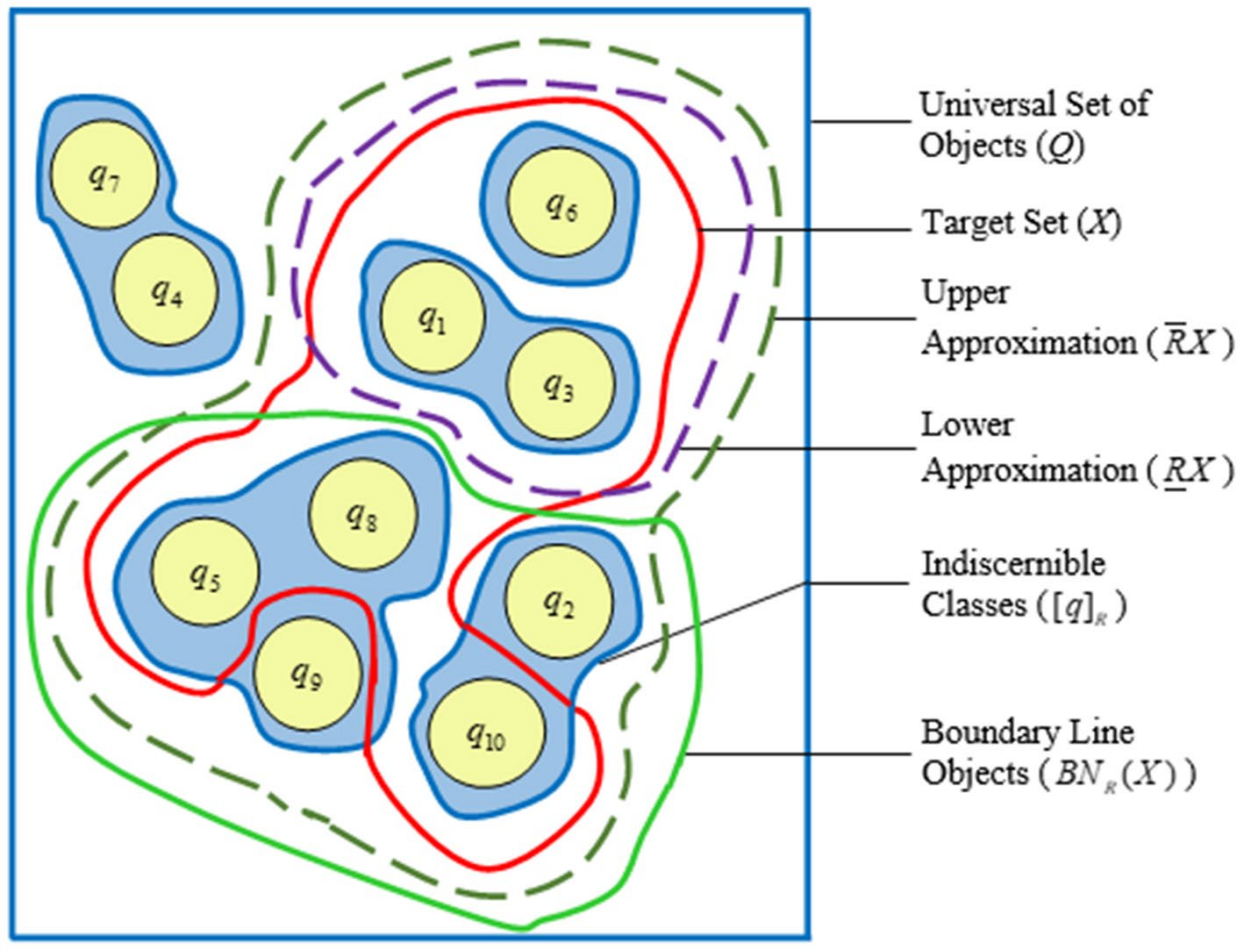
Representation of different rough set concepts.

We obtain $Q/R=\{\{q_1, q_3\}, \{q_2, q_{10}\}, \{q_4, q_7\},\{q_5, q_8, q_9\}, \{q_6\}\}$ by applying equivalence relations on the features $P = \{p_1, p_2, p_3, p_4, p_5\}$. Taking $X = \{q_1, q_3, q_5, q_6, q_8, q_{10}\}$ into account, we obtain ${\underline{R}}X = \{q_1, q_3, q_6\}$ and ${\overline{R}}X = \{q_1, q_2, q_3, q_5, q_6, q_8, \ q_9, q_{10}\}$. $BN_R(X) = \{q_2, q_5, q_8, q_9, q_{10}\}$ are the boundary line objects as a result. The boundary line portions, lower, and upper approximations of the RS are outlined in Fig. 1 from a broad perspective.

### Convictions of red deer optimization

Since ancient times, Scotland has supported populations of Red Deer (RD). Male stags and female hinds are the two main categories of RD. This animal exhibits extraordinary behavior when it is reproducing. Stags yell often loudly during this time of year to draw female hinds. Mostly, hind prefers males who yell frequently. The notions of the mating phenomenon are the foundation of Red Deer Optimization (RDO). It is a population-based meta-heuristics algorithm in which Male RDs (MRDs) have been chosen initially. Rest is regarded as a hind. MRD begins by roaring, then they split into two teams known as commanders and stags. Together, these two teams battle for control of the harem. Additionally, the quantity of hinds is related to the roaring and fighting skills of the commanders. As a result, in the harems, the commanders have numerous hindmattings. Further, a hind is mated by the closest male stags^6^.

Exploration and exploitation are two stages of the algorithm’s operation. The loudness of MRD in the search space promotes local search exploitation. Likewise, the manner in which combating between stags and commanders is taken into account in local searches to provide improved solutions. In the exploring stage, harems are also created and assigned to the commanders. The commanders mating with the hinds of the relevant harems and other harem during this phase. The matting phase of the algorithm, which creates RD offspring, is another stage of this optimization^35^.

Let us consider a population of RD’s defined in Eq. (7). Further, the fitness of each RD is obtained using Eq. (8), where *m* is the number of features.

7 $$\begin{aligned} q^{RD} = \{p_1, p_2, p_3, \cdots , p_m\} \end{aligned}$$

8 $$\begin{aligned} Fitness = f(q^{RD}) = f(p_1, p_2, p_3, \cdots , p_m) \end{aligned}$$

The procedure starts with an elementary population of size *n*. Further, the population is categorized into MRD and Hind RD (HRD). While HRDs are thought of as diversification, MRDs have intensification characteristics in the population. Besides, the MRDs enhance their ranks using the Eq. (9), where *UB* refers to upper bound and *LB* refers to lower bound of the search space. The constants $s_1$, $s_2$, and $s_3$ are the random numbers between 0 and 1 and refer to the three stages of roaring.

9 $$\begin{aligned} q_{new}^{MRD} = \left\{ \begin{array}{ccc} q_{old}^{MRD} + s_1\times ((UB - LB)\times s_2 + LB) &{} if &{} s_3\ge 0.5\\ &{} &{} \\ q_{old}^{MRD} - s_1\times ((UB - LB)\times s_2 + LB) &{} if &{} s_3 < 0.5 \end{array} \right. \end{aligned}$$

Further using Eq. (10), we calculate the number of commanders, where $\alpha \in [0, 1]$ refers to a random number and $N^{MRD}$ refers to the total number of MRDs. Similarly, $N^{Com}$ refers to the number of commanders. Equivalently, the number of stags $N^{Stag}$ is defined as $N^{Stag} = N^{MRD} - N^{Com}$.

10 $$\begin{aligned} N^{Com} = round(\alpha . N^{MRD}) \end{aligned}$$

The fighting behavior between commanders and stags that leads to two offspring is expressed analytically using Eqs. (11) and (12) respectively. Please take note that $b_1$ and $b_2$ are uniformly distributed random numbers between 0 and 1.

11 $$\begin{aligned} q_{new1} = \frac{(Com + Stag)}{2} + b_1\times ((UB-LB)\times b_2 + LB) \end{aligned}$$

12 $$\begin{aligned} q_{new2} = \frac{(Com + Stag)}{2} - b_1\times ((UB-LB)\times b_2 + LB) \end{aligned}$$

Further, it develops a harem. It is a herd of hinds that a male commander captured. The Objective Fitness (OF) of the male commander determines the number of hinds in a harem. Therefore, using $V_n = \nu _n - Max_{i}{\nu _i}$, hinds are distributed among commanders, where $\nu _n$ is the power of the $n^{th}$ commander and $V_n$ is its normalized value. Using Eq. (13), the normalized power of the commander is calculated.

13 $$\begin{aligned} Pow_{n} = \left| \frac{V_n}{\sum _{i=1}^{N^{Com}}V_i}\right| \end{aligned}$$

Consider $N^{Hind}$ is the total number of hinds, and $N-{n}^{harem} = round(Pow_{n}\times N^{Hind})$ can be used to calculate the number of hinds in a harem. Furthermore, a commander uses $N_{n}^{Harem_{mate}} = round(\delta _1 \cdot N_{n}^{Harem})$ to do the deer mating activity, where $\delta _1\in [0, 1]$ refers to the initial parameter concerning the percent of hinds are the parents in the same harem. The offspring produced by the mating process is defined in Eq. (14), where $c\in [0, 1]$ refers to a uniformly distributed random number.

14 $$\begin{aligned} q_{off} = \frac{(Com + Hind)}{2} + c\times (UB - LB) \end{aligned}$$

**Figure 2:**
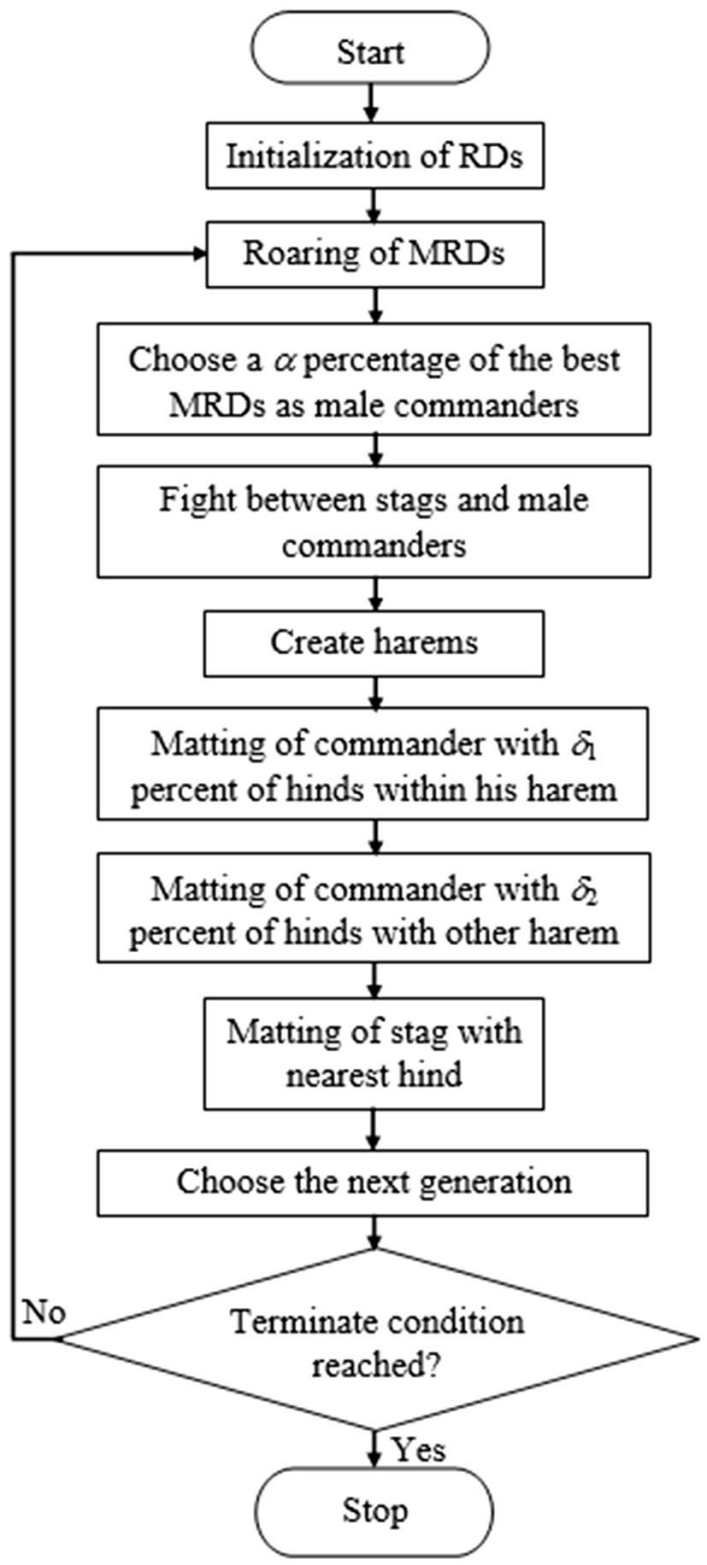
Flow diagram of RDO algorithm.

Moreover, a commander uses $N_{k}^{Harem_{mate}} = round(\delta _2 \cdot N_{k}^{Harem})$ to do the deer mating activity, where $\delta _2\in [0, 1]$ refers to the initial parameter concerning the percent of hinds are the parents in another harem. Here, *k* refers to a randomly chosen harem. Finally, the remained stag mates with the nearest hind. A distance function defined in Eq. (15) is used for a stag to find the nearest hind, where the distance between $i^{th}$ hind and a stag is denoted as $d_i$ and *J* refers to the dimensional space. The flow diagram of RDO is presented in Fig. 2.

15 $$\begin{aligned} d_i = \left\{ \sum _{j\in J}\left( q_j^{Stag} - q_j^{Hind_i}\right) ^2\right\} ^{1/2} \end{aligned}$$

## Overview of proposed research

**Figure 3:**
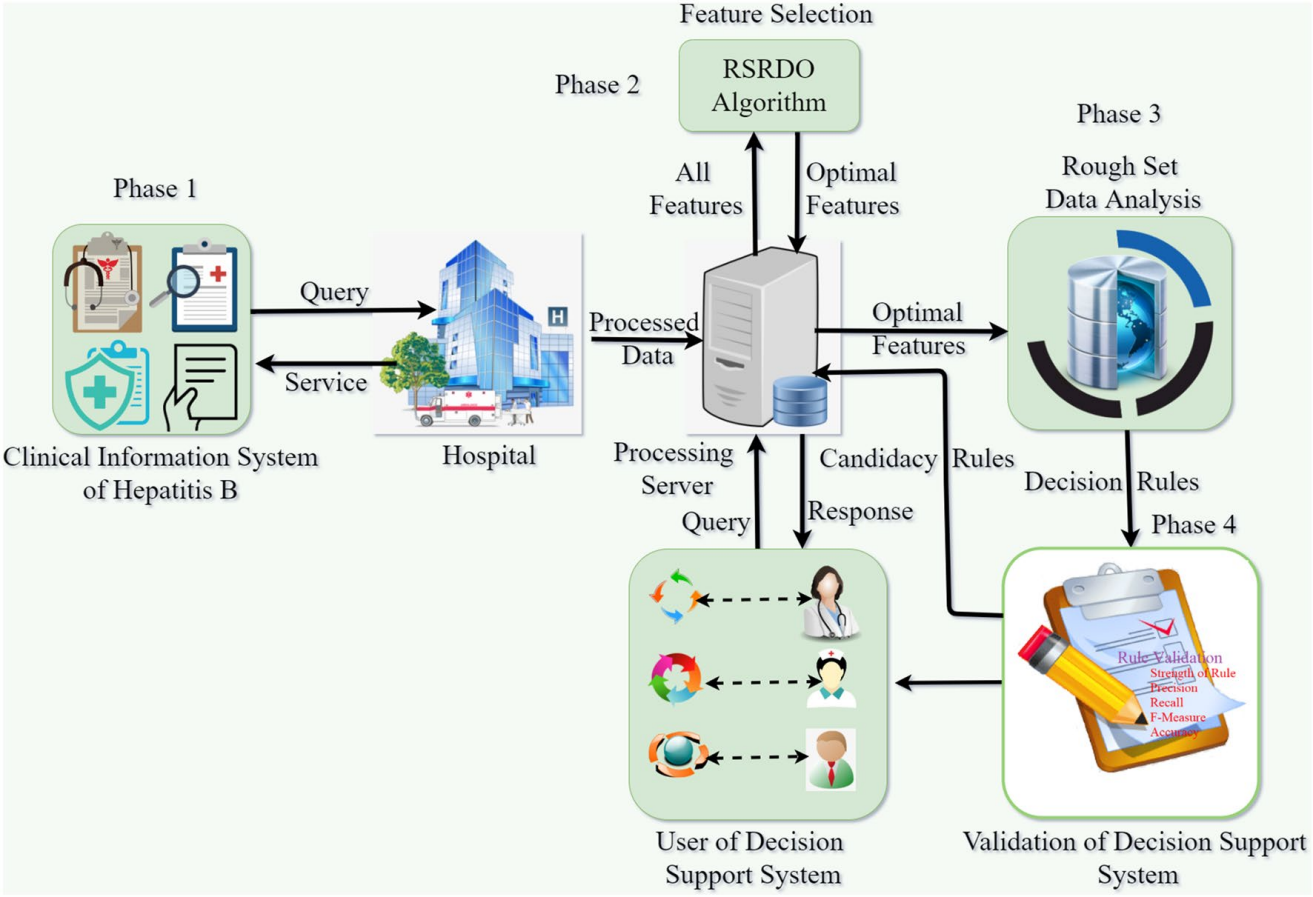
Graphical view of proposed research.

This section outlines the four-phased research design that is anticipated. At the early stage, a medicinal record system for hepatitis B is gathered. The medicinal record system demonstrates that independent of decisions, the conditional parameter values of different patients hold the same. Physicians’ differing opinions are the main reason and it ultimately results in uncertain data analysis. As clinical information systems involve uncertainty, uncovering hidden information can be difficult. As a result, when analyzing data, it is crucial to cope with ambiguous and partial information in classification. Hence, the main goal of this phase is to remove missing data and noise. The proposed Rough Set Red Deer Optimization (RSRDO) algorithm is used to further examine the processed decision system in the next phase. It aids in locating the prime features that influence the decision system. In the third phase, RS is used to develop the clinical information retrieval system. The decision rules are further validated during the fourth phase of the research design. Figure 3 displays the planned research’s block diagram.

In order to infer knowledge and develop a clinical information retrieval system, this study uses an integrated data analysis procedure that combines RS and Red Deer Optimization (RDO) algorithms. The proposed RSRDO controls the uncertainties that occur in the clinical decision system. Another goal of this study is to achieve high accuracy of classification with less number of conditional features. The medicinal record system for hepatitis B disease is used to build a clinical information retrieval system using the projected integrated technique RSRDO. We assume that a deer will find the best parameter subset given a binary bit string of length *m*. In this instance, *m* indicates the conditional parameters in the medicinal record system. If one of the component values in the solution vector is 1, the related conditional attribute is chosen. Similarly, if the component value is 0, the conditional attribute is not chosen for developing a clinical information retrieval system. Moreover, the fitness of each solution vector is determined using a fitness function as stated in Eq. (16).

16 $$\begin{aligned} Fitness\hspace{0.1cm} f(q) = \alpha \gamma (q) + \beta \left( 1 - \frac{m_{s}}{m_c}\right) \end{aligned}$$

In Eq. (16), the degree of dependency is referred to $\gamma (q)$ as described in Eq. (5). The terms “$m_s$” and “$m_c$” stand for selected parameters and total parameters correspondingly. The notion $\alpha$ refers to the degree of dependency whereas $\beta$ refers to the weight of other parameters considered. It is to be noted that $\alpha + \beta = 1$. Besides, the value of $\alpha$ must be high. The fitness function identifies the most relevant attributes pertaining to the disease hepatitis B. The procedure of the suggested RSRDO algorithm is defined in the earlier section.

The clinical information retrieval system for hepatitis B divides the condition into two groups: acute and chronic. This exploratory research uses the proposed RSRDO technique to find the important features, and RS to create a clinical information retrieval system. The clinical information retrieval system is further developed using RS based on the important feature values. So, the decision rules produced by using the integrated approach assist doctors in making the correct decision. Simultaneously, it saves the life of a person by saving time, and money.

The primary conditional features in the hepatitis B disease decision system are established using the RSRDO technique. Further, irrelevant features are eliminated from the decision table and decision rules are generated. It is also checked with domain experts that, the reduced decision system is suitable for building a clinical information retrieval system. The reduced medicinal record system is partitioned into two sections known as the training section and the testing section. A total of 70% of the data are in the training section, while 30% are in the testing section. The generation of RS decision rules is applied to the training section. Using Eq. (6), each decision rule’s accuracy is calculated. Moreover, each decision rule’s support, non-support, and strength are calculated. For the creation of a clinical information retrieval system, a threshold of 65% is considered. The decision rule whose accuracy is less than 65% is discarded.

The clinical information retrieval system is further examined using 30% of testing data in the validation phase. Various measures like recall (Recl.), precision (Precn.), accuracy (Acc.), and F-score are considered for obtaining the accuracy of the model. The F-score is computed to balance precision, and recall, and to analyze uneven data classification. These numerous measures are defined mathematically using Eqs. (17), (18), (19), and (20) respectively. It employs the terms $t^p$, $f^p$, $t^n$, and $f^n$ for true positive, false positive, true negative, and false negative respectively.

17 $$\begin{aligned} Recall\hspace{0.1cm} (Recl.) = \frac{|t^p|}{|t^p + f^n|} \end{aligned}$$

18 $$\begin{aligned} Precision\hspace{0.1cm} (Precn.) = \frac{|t^p|}{|t^p + f^p|} \end{aligned}$$

19 $$\begin{aligned} F-score = 2 \times \left( \frac{Precn. \times Recl.}{Precn. + Recl.}\right) \end{aligned}$$

20 $$\begin{aligned} Accuracy\hspace{0.1cm} (Acc.) = \frac{|t^p + t^n|}{|t^p + f^p + t^n + f^n|} \end{aligned}$$

## Experimental research on hepatitis B

**Figure 4:**
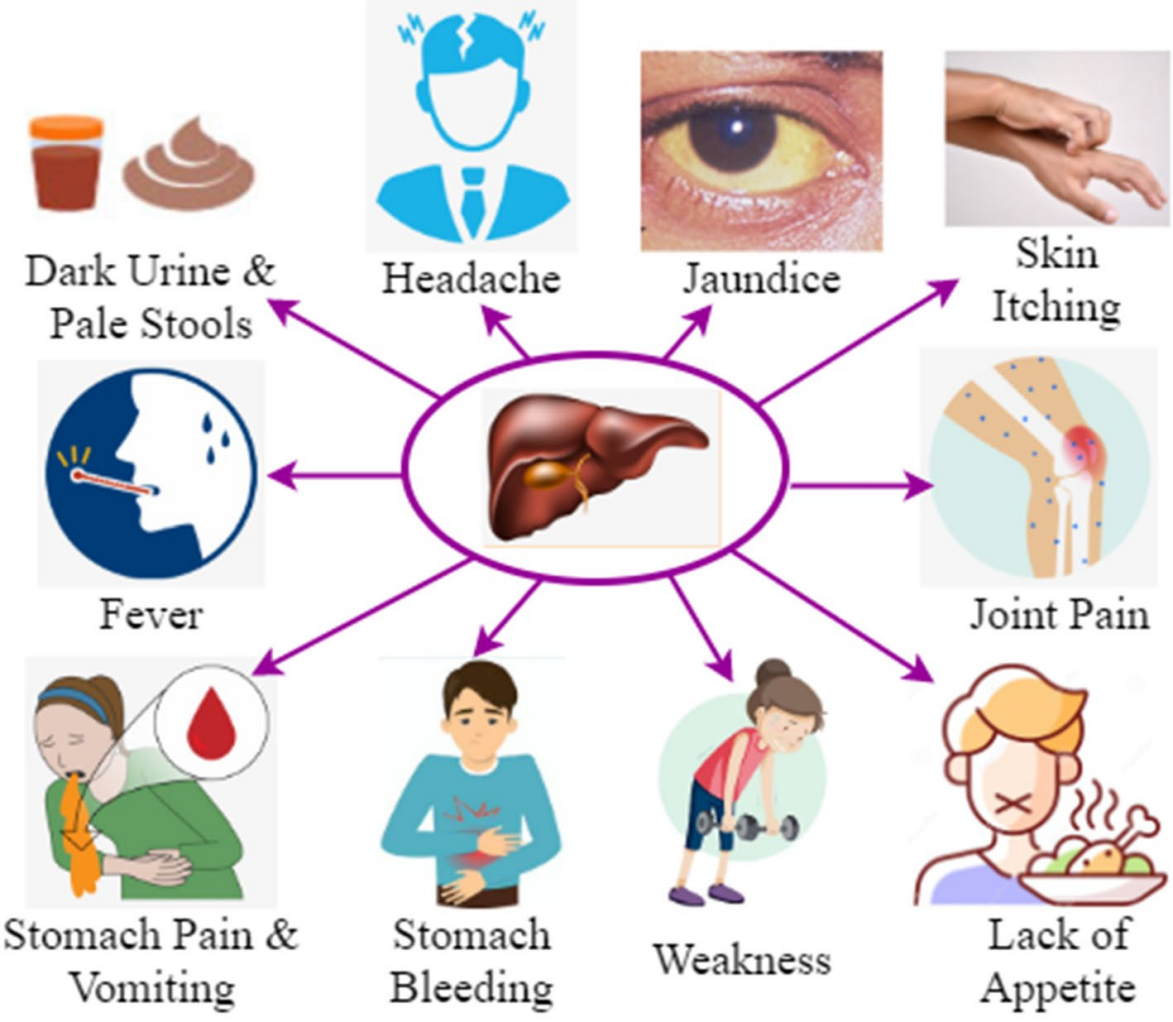
Symptoms of hepatitis B.

In this section, a clinical investigation of hepatitis B is described. The hepatitis B virus (HBV) is the cause of hepatitis B, a deadly liver illness. It significantly affects the state of world health. It can lead to persistent illness and significantly increases the risk of cirrhosis and liver cancer-related death. It is found in the liver. In addition to managing blood sugar levels and detoxifying the body, the liver also manages digestion, energy production, glycogen storage, and other bodily functions. Cells in the liver tissue are impacted by hepatitis, which compromises their functionality. Hepatitis A, B, C, D, and E are only a few of the several types. Hepatitis B is the most common liver infection, nonetheless, in the entire world. Using razors that have been used by an infected person, injecting drugs using an infected syringe, and contact with infectious bodily fluids are the main ways it is disseminated. Some of the common symptoms include jaundice, fever, skin itching, lack of appetite, weakness, ascites, abnormal blood clotting, dark urine, headache, pale stools, joint pain, and stomach bleeding. Figure 4 displays the hepatitis B signs and symptoms.

The features of the hepatitis B disease and its categories are listed in Table 2 below. Information for this data set was gathered from the UCI repository^36^. Additionally, medical records from some primary health centers in West Bengal, India, are taken into account for the analysis. It has one decision parameter $a_d$ with 19 conditional features $p_1, p_2, \ldots , p_{19}$. These conditional features are further categorized taking assistance from expert physicians. For instance, four groups of alk phosphate have been categorized: 26–96; 96.1–147; 147.1–194; and 194.1–295. These groups are nominated as 1, 2, 3, and 4 respectively for analysis. However, the data analysis is unaffected by this representation.

**Table 2:** Features of hepatitis B and its categorization.

Attribute	Classification	Attribute	Classification
Age ($p_1$)	$\ge 66$ V old (4)	Varices	No (0)
	45–65 old (3)	($p_{13}$)	Yes (1)
	25–45 mild (2)	Histology	No (0)
	7–25 young (1)	($p_{14}$)	Yes (1)
Gender $(p_2)$	Male (1)	Bilirubin	6–8 very high (4)
	Female (2)	($p_{15}$)	4–5.9 High (3)
Steroid ($p_3$)	No (0)		2–3.9 medium (2)
	Yes (1)		0.3–1.9 low (1)
Antivirals	No (0)	Alk phosphate	194.1–295; V high (4)
($p_4$)	Yes (1)	($p_{16}$)	147.1–194; high (3)
Fatigue ($p_5$)	No (0)		96.1–147; medium (2)
	Yes (1)		26–96; low (1)
Malaise ($p_6$)	No (0)	SGOT ($p_{17}$)	278.1–648; V high (4)
	Yes (1)		182.1–278; high (3)
Anorexia	No (0)		98.1–182; medium (2)
($p_7$)	Yes (1)		14–98; low (1)
Liver big ($p_8$)	No (0)	Albumin ($p_{18}$)	4.9–6.4; high (3)
	Yes (1)		3.6–4.8; medium (2)
Liver firm	No (0)		2.1–3.5; low (1)
($p_9$) Spleen palpable	Yes (1)		
	No (0)	Protime ($p_{19}$)	78.1–100; V high (4)
($p_{10}$)	Yes (1)		58.1–78; high (3)
Spiders ($p_{11}$)	No (0)		39.1–58; medium (2)
	Yes (1)		0-39; low (1)
Ascites ($p_{12}$)	No (0)	Hepatitis ($p_d$)	Chronic (0)
	Yes (1)		Acute (1)

The hepatitis B medicinal records are divided into two classifications, chronic and acute, according to the judgment. In this experimental study to create a clinical information retrieval system, an integrated RSRDO approach is used. Based on the values of the conditional features, a specific judgment is taken into consideration for each patient. Therefore, using the RSRDO feature selection algorithm and rough set is crucial to produce decision rules. This aids the clinical information retrieval system in making a preliminary diagnosis of an illness. A sample medicinal record system is illustrated in Table 3.

**Table 3:** Medicinal record system of hepatitis B disease.

Records	$p_1$	$p_2$	$p_3$	$p_4$	$p_5$	$p_6$	$p_7$	$p_8$	$p_9$	$p_{10}$
$q_1$	1	1	1	1	1	0	0	1	0	0
$q_2$	1	2	1	0	1	1	1	0	1	1
$q_3$	2	2	0	1	1	1	0	1	1	0
$q_4$	2	2	1	0	1	0	0	1	0	1
$q_5$	3	1	0	0	1	0	0	1	0	1
$q_6$	4	1	1	0	1	1	0	1	1	0
$q_7$	3	2	1	0	1	0	0	1	1	1
$q_8$	3	2	0	0	1	1	0	1	0	0
$q_9$	3	2	0	0	1	1	0	1	1	1
$q_{10}$	3	1	1	0	1	0	0	1	1	0

Records	$p_{11}$	$p_{12}$	$p_{13}$	$p_{14}$	$p_{15}$	$p_{16}$	$p_{17}$	$p_{18}$	$p_{19}$	$p_{d}$
$q_1$	0	0	0	0	1	1	1	2	3	1
$q_2$	1	0	0	0	1	2	1	2	1	1
$q_3$	0	0	0	0	2	4	1	2	2	0
$q_4$	1	1	0	1	1	2	1	1	2	0
$q_5$	1	0	1	1	1	1	1	2	2	1
$q_6$	1	0	0	0	1	2	2	2	2	1
$q_7$	0	1	1	1	2	3	1	1	1	0
$q_8$	1	1	0	1	3	1	1	1	1	0
$q_9$	1	0	0	1	1	2	2	2	1	0
$q_{10}$	1	0	0	0	1	3	1	1	1	1

## Result analysis of proposed model

**Figure 5:**
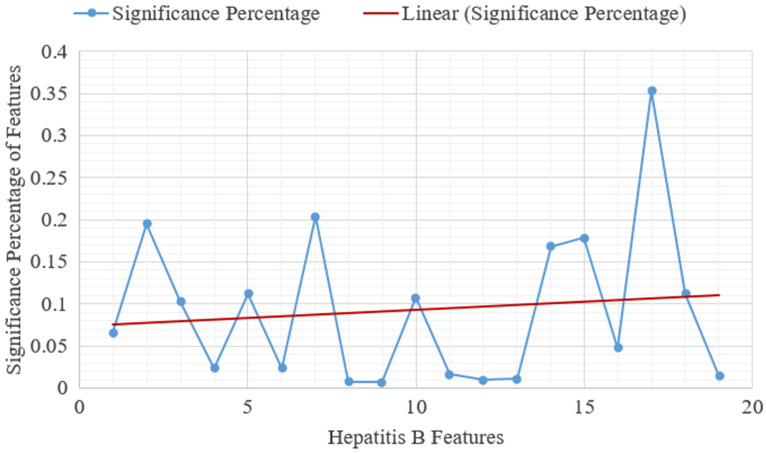
Significance of characteristics of hepatitis B.

The investigation is conducted using a computer system with an Intel Core i5-4200U CPU running at 1.60 GHz and 2.30 GHz, Windows 10, and 32GB of RAM. The evaluation of the investigation is done using Python. Furthermore, the proposed RSRDO procedure is used to find the significant features in the data of 643 patients. A total of 1000 iterations are taken into account for the investigation. Besides, we have considered 10 runs and each feature’s significance is calculated. Further, we have computed the average of all the runs to get the accuracy of each feature. The significance of each feature for all 10 runs is presented in Table 4. The primary characteristic is the total number of characteristics with significance values over the trend line. Figure 5 exhibits the feature’s significance relating to the proposed RSRDO algorithm. Nine features in all have been chosen for analysis. Gender ($p_2$), steroid ($p_3$), fatigue ($p_5$), anorexia ($p_7$), palpable spleen ($p_{10}$), histology ($p_{14}$), bilirubin ($p_{15}$), SGOT ($p_{17}$), and albumin ($p_{18}$) are the indicated features. The information system also removes other features like age ($p_1$), antivirals ($p_4$), malaise ($p_6$), liver big ($p_8$), liver firm ($p_9$), spiders ($p_{11}$), ascites ($p_{12}$), varices ($p_{13}$), alk phosphate ($p_{16}$), and protime ($p_{19}$).

**Table 4:** Significance of characteristics in each run.

Run 1	Run 2	Run 3	Run 4	Run 5	Run 6	Run 7	Run 8	Run 9	Run 10	Average
0.60204	0.02284	0.00742	0.0119	0.00395	0.00198	0.002	0.00079	0.00402	0.00401	0.06609
0.00456	0.00548	0.00296	0.00472	0.01702	0.02321	0.00211	0.13497	0.7716	0.98544	0.19521
0.01268	0.00885	0.00994	0.01238	0.93777	0.01119	0.00451	0.0008	0.00427	0.02174	0.10241
0.00553	0.00975	0.01734	0.00497	0.00306	0.03097	0.00166	0.03719	0.00399	0.12116	0.02356
0.00385	0.00447	0.02415	0.9944	0.04556	0.0222	0.00492	0.00252	0.00772	0.00864	0.11184
0.00535	0.01042	0.01357	0.03369	0.05805	0.00283	0.09234	0.00603	0.00295	0.01238	0.02376
0.00316	0.99232	0.00986	0.0041	0.01291	0.99846	0.00648	0.00222	0.00452	0.00664	0.20407
0.00597	0.00618	0.00724	0.01301	0.01636	0.00224	0.00526	0.00429	0.01212	0.00706	0.00797
0.00479	0.01272	0.01555	0.00285	0.01334	0.00286	0.00252	0.00221	0.00511	0.00912	0.00711
0.99494	0.00863	0.01495	0.02232	0.00914	0.00341	0.00314	0.00369	0.00879	0.00764	0.10767
0.05962	0.00498	0.00393	0.05721	0.0069	0.00455	0.0041	0.01282	0.004	0.0077	0.01658
0.00427	0.03853	0.0048	0.00321	0.0112	0.01504	0.00176	0.00778	0.00157	0.00874	0.00969
0.03006	0.01527	0.018	0.00369	0.01411	0.01183	0.00563	0.00421	0.00326	0.00274	0.01088
0.00966	0.79231	0.83853	0.00749	0.00578	0.0107	0.0041	0.00889	0.0023	0.00694	0.16867
0.00487	0.05572	0.00478	0.93698	0.07023	0.5004	0.0029	0.00192	0.1387	0.06762	0.17841
0.00672	0.00671	0.00624	0.03024	0.00537	0.0702	0.0483	0.15515	0.13608	0.00913	0.04741
0.01636	0.02959	0.98749	0.08703	0.24797	0.11714	0.04057	0.9964	0.00923	0.9931	0.35249
0.01013	0.0051	0.02482	0.01317	0.98815	0.05441	0.00777	0.00386	0.00535	0.00611	0.11189
0.00599	0.01693	0.01438	0.01934	0.00534	0.00255	0.0018	0.00523	0.0056	0.05945	0.01366
0.00415	0.01545	0.01204	0.01592	0.00223	0.00219	0.00131	0.00259	0.00472	0.007	0.00676

The reduced medicinal record system is split into 70% (450) training data and 30% (193) testing data for building a clinical information retrieval system making use of the RS. The training set includes 137 acute instances and 313 chronic instances of hepatitis B. The RS technique is imposed to investigate these 450 training data for generating decision rules. The hepatitis B decision system’s decision rules, which were created from training data, are shown in Tables 5 and 6 respectively. Decision rules that have an accuracy rate of less than 65% are discarded as candidate rules. Furthermore, these decision rules are validated by making use of 193 testing data.

**Table 5:** Training decision rules of hepatitis B confining proposed RSRDO.

Rule No.	Narration of decision rule	Supp.	Non-support	Strength ($\%$)	Accy. ($\%$)
1	$p_2 = 1, p_3 = 0, p_{10} = 0, p_{14} = 0, p_{18} = 1 \rightarrow p_d = 0$	10	1	11.11	90.91
2	$p_3 = 1, p_7 = 0, p_{14}= 0, p_{18} = 1 \rightarrow p_d = 0$	9	1	10.00	90.00
3	$p_{15} = 3, p_{18} = 1 \rightarrow p_d = 0$	6	1	6.67	85.71
4	$p_{15} = 4, p_{18} = 2 \rightarrow p_d = 0$	7	1	7.78	87.50
5	$p_2 = 2, p_3 = 0, p_5 = 1, p_{10} = 0, p_{18} = 2 \rightarrow p_d = 0$	5	1	5.56	83.33
6	$p_2 = 1, p_3 = 1, p_7 = 1, p_{15} = 2, p_{18} = 1 \rightarrow p_d = 0$	5	0	5.56	100
7	$p_2 = 1, p_3 = 0, p_7 = 1, p_{10} = 0, p_{17} = 2 \rightarrow p_d = 0$	5	0	5.56	100
8	$p_2 = 2, p_5 = 1, p_{10} = 1, p_{14} = 0, p_{17} = 2 \rightarrow p_d = 0$	7	1	7.78	87.50
9	$p_7 = 1, p_{14} = 1, p_{15} = 3, p_{18} = 2 \rightarrow p_d = 0$	9	2	10.00	81.82
10	$p_3 = 1, p_{10} = 0, p_{14} = 1, p_{17} = 3 \rightarrow p_d = 0$	6	2	6.67	75.00
11	$p_{14} = 1, p_{15} = 2, p_{17} = 2, p_{18} = 2 \rightarrow p_d = 0$	8	0	8.89	100
12	$p_2 = 2, p_5 = 1, p_{14} = 0, p_{15} = 2, p_{18} = 3 \rightarrow p_d = 0$	3	0	3.33	100
13	$p_2 = 1, p_3 = 0, p_5 = 0, p_{15} = 3, p_{18} = 3 \rightarrow p_d = 0$	6	0	6.67	100
14	$p_3 = 1, p_7 = 0, p_{14} = 0, p_{15} = 3 \rightarrow p_d = 0$	5	0	5.56	100
15	$p_2 = 2, p_{10} = 1, p_{14} = 1, p_{17} = 3 \rightarrow p_d = 0$	7	0	7.78	100
16	$p_2 = 2, p_5 = 0, p_{14} = 1 \rightarrow p_d = 1$	2	4	0.56	33.33
17	$p_2 = 1, p_5 = 1, p_{14} = 1 \rightarrow p_d = 1$	3	2	0.83	60.00
18	$p_2 = 1, p_3 = 1, p_5 = 1, p_{15} = 1 \rightarrow p_d = 1$	3	0	0.83	100
19	$p_3 = 0, p_{10} = 1, p_{14} = 1, p_{15} = 2, p_{18} = 3 \rightarrow p_d = 1$	5	1	1.39	83.33
20	$p_3 = 0, p_5 = 1, p_{10} = 0, p_{15} = 1, p_{18} = 3 \rightarrow p_d = 1$	7	1	1.94	87.50
21	$p_5 = 0, p_7 = 0, p_{10}= 1, p_{15} = 1, p_{18} = 2 \rightarrow p_d = 1$	5	0	1.39	100
22	$p_2 = 1, p_7 = 1, p_{10} = 1, p_{15} = 1, p_{17} = 2 \rightarrow p_d = 1$	4	0	1.11	100
23	$p_{10} = 1, p_{14} = 1, p_{17} = 2, p_{18} = 3 \rightarrow p_d = 1$	6	0	1.67	100
24	$p_5 = 0, p_7 = 0, p_{10} = 1, p_{15} = 2, p_{18} = 3 \rightarrow p_d = 1$	4	1	1.11	80.00
25	$p_2 = 1, p_7 = 1, p_{14} = 0, p_{17} = 3 \rightarrow p_d = 1$	2	0	0.56	100
26	$p_7 = 0, p_{10} = 0, p_{14} = 0, p_{17} = 2, p_{18} = 3 \rightarrow p_d = 1$	2	0	0.56	100
27	$p_5 = 1, p_7 = 1, p_{10} = 0, p_{17} = 1, p_{18} = 3 \rightarrow p_d = 1$	5	0	1.39	100
28	$p_3 = 1, p_5 = 0, p_{10} = 0, p_{14} = 1, p_{17} = 1 \rightarrow p_d = 1$	5	0	1.39	100
29	$p_2 = 2, p_5 = 0, p_{15} = 2 \rightarrow p_d = 1$	7	1	1.94	87.50
30	$p_2 = 2, p_5 = 0, p_{18} = 2 \rightarrow p_d = 1$	4	3	1.11	57.14
31	$p_7 = 1, p_{10} = 1, p_{14} = 0, p_{15} = 2, p_{17} = 2 \rightarrow p_d = 1$	3	0	0.83	100
32	$p_2 = 2, p_{14} = 1, p_{15} = 3 \rightarrow p_d = 1$	9	3	2.50	75.00
33	$p_2 = 1, p_{17 }= 3, p_{18 }= 2 \rightarrow p_d = 1$	6	1	1.67	85.71
34	$p_5 = 1, p_{10} = 0, p_{17} = 4 \rightarrow p_d = 1$	4	0	1.11	100
35	$p_3 = 0, p_{10} = 1, p_{15} = 2, p_{18} = 1 \rightarrow p_d = 1$	3	0	0.83	100
36	$p_3 = 1, p_{10} = 0, p_{14} = 0, p_{17} = 3 \rightarrow p_d = 1$	2	0	0.56	100
37	$p_2 = 2, p_3 = 0, p_7 = 1, p_{10} = 1\rightarrow p_d = 1$	2	0	0.56	100
38	$p_2 = 1, p_3 = 1, p_{10} = 0, p_{18} = 2 \rightarrow p_d = 1$	8	1	2.22	88.89
39	$p_{15} = 2, p_{17} = 2, p_{18} = 3 \rightarrow p_d = 1$	7	1	1.94	87.50
40	$p_5 = 1, p_{18} = 1 \rightarrow p_d = 1$	5	0	1.39	100
41	$p_2 = 2, p_{14} = 0, p_{15} = 2, p_{18} = 2 \rightarrow p_d = 1$	9	1	2.50	90.00
42	$p_{14} = 1, p_{15} = 2, p_{17} = 1, p_{18} = 1 \rightarrow p_d = 1$	5	0	1.39	100
43	$p_3 = 0, p_7 = 0, p_{17} = 3, p_{18} = 3 \rightarrow p_d = 1$	3	0	0.83	100
44	$p_7 = 0, p_{14} = 1, p_{15} = 3, p_{18}=2 \rightarrow p_d = 1$	3	1	0.83	75.00
45	$p_7 = 1, p_{10} = 0, p_{14} = 0, p_{17} = 2, p_{18} = 3 \rightarrow p_d = 0$	7	1	7.78	87.50
46	$p_3 = 1, p_7 = 1, p_{10} = 0, p_{17} = 2, p_{18} = 2 \rightarrow p_d = 1$	8	0	2.22	100
47	$p_7 = 1, p_{14} = 1, p_{17} = 1, p_{18} = 1 \rightarrow p_d = 1$	2	0	0.56	100
48	$p_7 = 1, p_{10} = 0, p_{14} = 1, p_{18} = 3 \rightarrow p_d = 1$	5	2	1.39	71.43
49	$p_5 = 0, p_{10} = 1, p_{17} = 4 \rightarrow p_d = 1$	3	1	0.83	75.00

**Table 6:** Training decision rules of hepatitis B confining proposed RSRDO (continued).

Rule No.	Narration of decision rule	Supp.	Non-support	Strength (%)	Accy. (%)
50	$p_2 = 1, p_7 = 1, p_{10} = 0, p_{14} = 1, p_{17} = 1 \rightarrow p_d = 1$	9	0	2.50	100
51	$p_3 = 1, p_{10} = 0, p_{15} = 2, p_{18} = 3 \rightarrow p_d = 1$	5	0	1.39	100
52	$p_{17} = 4, p_{18} = 3 \rightarrow p_d = 1$	7	3	1.94	70.00
53	$p_3 = 0, p_{15} = 1, p_{17} = 2, p_{18} = 1 \rightarrow p_d = 1$	4	3	1.11	57.14
54	$p_2 = 1, p_7 = 1, p_{15} = 3, p_{17} = 1, p_{18} = 2 \rightarrow p_d = 1$	6	2	1.67	75.00
55	$p_3 = 0, p_7 = 0, p_{10} = 1, p_{17} = 1, p_{18} = 3 \rightarrow p_d = 1$	5	0	1.39	100
56	$p_2 = 1, p_7 = 0, p_{10} = 0, p_{17} = 2, p_{18} = 2 \rightarrow p_d = 1$	7	0	1.94	100
57	$p_3 = 1, p_{14} = 1, p_{15} = 2, p_{18} = 2 \rightarrow p_d = 1$	6	1	1.67	85.71
58	$p_3 = 1, p_5 = 1, p_7 = 0, p_{14} = 1, p_{15} = 1, p_{17} = 2 \rightarrow p_d = 0$	4	0	4.44	100
59	$p_2 = 2, p_3 = 0, p_5 = 1, p_{10} = 0, p_{15} = 2, p_{17} = 1 \rightarrow p_d = 0$	5	3	5.56	62.50
60	$p_2 = 1, p_3 = 0, p_5 = 0, p_{10} = 0, p_{14} = 1, p_{18} = 3 \rightarrow p_d = 0$	5	2	5.56	71.43
61	$p_2 = 2, p_3 = 1, p_5 = 1, p_{10} = 1, p_{15} = 1, p_{18} = 2 \rightarrow p_d = 0$	8	0	8.89	100
62	$p_2 = 1, p_3 = 0, p_5 = 0, p_{10} = 0, p_{15} = 1, p_{18} = 2 \rightarrow p_d = 0$	4	1	4.44	80.00
63	$p_2 = 2, p_5 = 1, p_{10} = 0, p_{15} = 1, p_{17} = 1, p_{18} = 2 \rightarrow p_d = 0$	6	1	6.67	85.71
64	$p_2 = 2, p_7 = 1, p_{10} = 1, p_{15} = 1, p_{17} = 1, p_{18} = 3 \rightarrow p_d = 0$	5	0	5.56	100
65	$p_3 = 1, p_5 = 0, p_{10} = 0, p_{14} = 0, p_{17} = 1, p_{18} = 3 \rightarrow p_d = 0$	5	1	5.56	83.33
66	$p_3 = 0, p_5 = 0, p_{10} = 1, p_{14} = 0, p_{15}=2, p_{17}=1, p_{18} = 2 \rightarrow p_d = 0$	8	1	8.89	88.89
67	$p_2 = 1, p_3 = 0, p_5 = 1, p_{14} = 0, p_{15} = 1, p_{17} = 1, p_{18} = 2 \rightarrow p_d = 0$	3	1	3.33	75.00

It shows from the data used for training, 67 decision rules were generated. It is evident from Tables 5 and 6 that there are 5 rules that are less accurate than the specified conception value 65%. Hence, these 5 rules are removed. Further, 62 decision rules are analyzed using 193 (30%) medical records. It includes 84 records of acute cases and 109 records of chronic cases. The testing analysis is presented in Tables 7 and 8 respectively.

**Table 7:** Testing decision rules of hepatitis B confining proposed RSRDO.

Rule No.	Narration of decision rule	Supp.	Non-support	Strength (%)	Accy. (%)
1	$p_2 = 1, p_3 = 0, p_{10} = 0, p_{14} = 0, p_{18} = 1 \rightarrow p_d = 0$	3	1	5.08	75.00
2	$p_3 = 1, p_7 = 0, p_{14}= 0, p_{18} = 1 \rightarrow p_d = 0$	3	0	5.08	100.0
3	$p_{15} = 3, p_{18} = 1 \rightarrow p_d = 0$	2	2	3.39	50.00
4	$p_{15} = 4, p_{18} = 2 \rightarrow p_d = 0$	3	1	5.08	75.00
5	$p_2 = 2, p_3 = 0, p_5 = 1, p_{10} = 0, p_{18} = 2 \rightarrow p_d = 0$	2	0	3.39	100.0
6	$p_2 = 1, p_3 = 1, p_7 = 1, p_{15} = 2, p_{18} = 1 \rightarrow p_d = 0$	2	1	3.39	66.67
7	$p_2 = 1, p_3 = 0, p_7 = 1, p_{10} = 0, p_{17} = 2 \rightarrow p_d = 0$	3	0	5.08	100.0
8	$p_2 = 2, p_5 = 1, p_{10} = 1, p_{14} = 0, p_{17} = 2 \rightarrow p_d = 0$	2	1	3.39	66.67
9	$p_7 = 1, p_{14} = 1, p_{15} = 3, p_{18} = 2 \rightarrow p_d = 0$	2	2	3.39	50.00
10	$p_3 = 1, p_{10} = 0, p_{14} = 1, p_{17} = 3 \rightarrow p_d = 0$	2	2	3.39	50.00
11	$p_{14} = 1, p_{15} = 2, p_{17} = 2, p_{18} = 2 \rightarrow p_d = 0$	1	0	1.69	100.0
12	$p_2 = 2, p_5 = 1, p_{14} = 0, p_{15} = 2, p_{18} = 3 \rightarrow p_d = 0$	2	0	3.39	100.0
13	$p_2 = 1, p_3 = 0, p_5 = 0, p_{15} = 3, p_{18} = 3 \rightarrow p_d = 0$	3	0	5.08	100.0
14	$p_3 = 1, p_7 = 0, p_{14} = 0, p_{15} = 3 \rightarrow p_d = 0$	2	0	3.39	100.0
15	$p_2 = 2, p_{10} = 1, p_{14} = 1, p_{17} = 3 \rightarrow p_d = 0$	3	0	5.08	100.0
18	$p_2 = 1, p_3 = 1, p_5 = 1, p_{15} = 1 \rightarrow p_d = 1$	4	0	2.99	100.0
19	$p_3 = 0, p_{10} = 1, p_{14} = 1, p_{15} = 2, p_{18} = 3 \rightarrow p_d = 1$	3	2	2.24	60.00
20	$p_3 = 0, p_5 = 1, p_{10} = 0, p_{15} = 1, p_{18} = 3 \rightarrow p_d = 1$	5	1	3.73	83.33
21	$p_5 = 0, p_7 = 0, p_{10}= 1, p_{15} = 1, p_{18} = 2 \rightarrow p_d = 1$	4	0	2.99	100.0
22	$p_2 = 1, p_7 = 1, p_{10} = 1, p_{15} = 1, p_{17} = 2 \rightarrow p_d = 1$	4	0	2.99	100.0
23	$p_{10} = 1, p_{14} = 1, p_{17} = 2, p_{18} = 3 \rightarrow p_d = 1$	3	0	2.24	100.0
24	$p_5 = 0, p_7 = 0, p_{10} = 1, p_{15} = 2, p_{18} = 3 \rightarrow p_d = 1$	5	2	3.73	71.43
25	$p_2 = 1, p_7 = 1, p_{14} = 0, p_{17} = 3 \rightarrow p_d = 1$	3	0	2.24	100.0
26	$p_7 = 0, p_{10} = 0, p_{14} = 0, p_{17} = 2, p_{18} = 3 \rightarrow p_d = 1$	3	0	2.24	100.0
27	$p_5 = 1, p_7 = 1, p_{10} = 0, p_{17} = 1, p_{18} = 3 \rightarrow p_d = 1$	3	0	2.24	100.0
28	$p_3 = 1, p_5 = 0, p_{10} = 0, p_{14} = 1, p_{17} = 1 \rightarrow p_d = 1$	3	0	2.24	100.0
29	$p_2 = 2, p_5 = 0, p_{15} = 2 \rightarrow p_d = 1$	4	3	2.99	57.14

**Table 8:** Testing decision rules of hepatitis B confining proposed RSRDO (continued).

Rule No.	Narration of decision rule	Supp.	Non-support	Strength (%)	Accy. (%)
31	$p_7 = 1, p_{10} = 1, p_{14} = 0, p_{15} = 2, p_{17} = 2 \rightarrow p_d = 1$	4	0	2.99	100.0
32	$p_2 = 2, p_{14} = 1, p_{15} = 3 \rightarrow p_d = 1$	3	2	2.24	60.00
33	$p_2 = 1, p_{17 }= 3, p_{18 }= 2 \rightarrow p_d = 1$	5	1	3.73	83.33
34	$p_5 = 1, p_{10} = 0, p_{17} = 4 \rightarrow p_d = 1$	3	0	2.24	100.0
35	$p_3 = 0, p_{10} = 1, p_{15} = 2, p_{18} = 1 \rightarrow p_d = 1$	3	0	2.24	100.0
36	$p_3 = 1, p_{10} = 0, p_{14} = 0, p_{17} = 3 \rightarrow p_d = 1$	4	0	2.99	100.0
37	$p_2 = 2, p_3 = 0, p_7 = 1, p_{10} = 1\rightarrow p_d = 1$	3	0	2.24	100.0
38	$p_2 = 1, p_3 = 1, p_{10} = 0, p_{18} = 2 \rightarrow p_d = 1$	2	1	1.49	66.67
39	$p_{15} = 2, p_{17} = 2, p_{18} = 3 \rightarrow p_d = 1$	3	1	2.24	75.00
40	$p_5 = 1, p_{18} = 1 \rightarrow p_d = 1$	3	0	2.24	100.0
41	$p_2 = 2, p_{14} = 0, p_{15} = 2, p_{18} = 2 \rightarrow p_d = 1$	5	1	3.73	83.33
42	$p_{14} = 1, p_{15} = 2, p_{17} = 1, p_{18} = 1 \rightarrow p_d = 1$	4	0	2.99	100.0
43	$p_3 = 0, p_7 = 0, p_{17} = 3, p_{18} = 3 \rightarrow p_d = 1$	4	0	2.99	100.0
44	$p_7 = 0, p_{14} = 1, p_{15} = 3, p_{18}=2 \rightarrow p_d = 1$	5	3	3.73	62.50
45	$p_7 = 1, p_{10} = 0, p_{14} = 0, p_{17} = 2, p_{18} = 3 \rightarrow p_d = 0$	3	1	5.08	75.00
58	$p_3 = 1, p_5 = 1, p_7 = 0, p_{14} = 1, p_{15} = 1, p_{17} = 2 \rightarrow p_d = 0$	2	0	3.39	100.0
60	$p_2 = 1, p_3 = 0, p_5 = 0, p_{10} = 0, p_{14} = 1, p_{18} = 3 \rightarrow p_d = 0$	2	2	3.39	50.00
61	$p_2 = 2, p_3 = 1, p_5 = 1, p_{10} = 1, p_{15} = 1, p_{18} = 2 \rightarrow p_d = 0$	2	0	3.39	100.0
62	$p_2 = 1, p_3 = 0, p_5 = 0, p_{10} = 0, p_{15} = 1, p_{18} = 2 \rightarrow p_d = 0$	3	2	5.08	60.00
63	$p_2 = 2, p_5 = 1, p_{10} = 0, p_{15} = 1, p_{17} = 1, p_{18} = 2 \rightarrow p_d = 0$	3	1	5.08	75.00
64	$p_2 = 2, p_7 = 1, p_{10} = 1, p_{15} = 1, p_{17} = 1, p_{18} = 3 \rightarrow p_d = 0$	3	1	5.08	75.00
65	$p_3 = 1, p_5 = 0, p_{10} = 0, p_{14} = 0, p_{17} = 1, p_{18} = 3 \rightarrow p_d = 0$	2	1	3.39	66.67
66	$p_3 = 0, p_5 = 0, p_{10} = 1, p_{14} = 0, p_{15}=2, p_{17}=1, p_{18} = 2 \rightarrow p_d = 0$	3	1	5.08	75.00
67	$p_2 = 1, p_3 = 0, p_5 = 1, p_{14} = 0, p_{15} = 1, p_{17} = 1, p_{18} = 2 \rightarrow p_d = 0$	1	0	1.69	100.0

Tables 7 and 8 show that the rules 3, 9, 10, 19, 29, 32, 44, 60, and 62 are removed because of accuracy lower than 65%. It is evident that decision rules are reduced by 14.52% as a result of the testing study. The confusion matrix is also developed in order to assess the correctness of the suggested RSRDO procedure. The confusion matrix for the RSRDO procedure is shown in Table 9. It is seen that the RSRDO procedure has acquired a 91.7% accuracy level.

**Table 9:** Performance measure of RSRDO over hepatitis B disease.

Description	$t^n$	$t^p$	$f^n$	$f^p$	Recl. (%)	Precn. (%)	F-Measure (%)	Acc. (%)
$p_d=0$	84	95	5	9	95.0	91.3	93.1	92.7
$p_d=1$	109	66	9	9	88.0	88.0	88.0	90.7
Total	193	161	14	18	92.0	89.9	90.9	91.7

## Comparison analysis

This section conducts a comparison of the RSRDO procedure with the Decision Tree (DT) procedure, RS procedure, and Red Deer Optimization—Decision Tree (RDODT) procedure. The individual comparison analysis is shown in the subsection that follows.

### Comparison analysis with RS

Making use of 450 patient records of the training data, RS data analysis is performed, which takes into account all hepatitis B features. 34 decision rules are generated from the RS data analysis^37^. Since every rule has a score of at least 65%, it is regarded as a candidate rule. The decisions created using the RS are shown in Table 10. It shows that the RS model produces 6% more rules than the RSRDO model.

**Table 10:** Training decision rules of hepatitis B confining RS.

Rule No.	Narration of decision rule	Supp.	Non-support	Strength (%)	Accy. (%)
1	$p_{11}=0, p_{16}=1, p_{18}=2 \Rightarrow p_d=1$	11	1	31.3	91.6
2	$p_{11}=0, p_{12}=0, p_{16}=1 \Rightarrow p_d=1$	15	2	38.8	88.2
3	$p_9=0, p_{11}=0, p_{18}=2 \Rightarrow p_d=1$	5	0	30.8	100
4	$p_9=0, p_{11}=0, p_{12}=0 \Rightarrow p_d=1$	17	2	42.7	89.4
5	$p_6=0, p_{15}=1, p_{16}=1 \Rightarrow p_d=1$	13	1	36.2	92.8
6	$p_6=0, p_{13}=0, p_{11}=0, p_{16}=1 \Rightarrow p_d=1$	7	2	49.1	77.7
7	$p_6=0, p_9=0, p_{11}=0, p_{16}=1 \Rightarrow p_d=1$	9	1	42.7	90.0
8	$p_3=1, p_{13}=0, p_{18}=2 \Rightarrow p_d=1$	9	0	32.8	100
9	$p_3=1, p_{13}=0, p_{16}=2 \Rightarrow p_d=1$	11	0	32.8	100
10	$p_3=1, p_{12}=0, p_{15}=3 \Rightarrow p_d=1$	14	2	41.2	87.5
11	$p_3=1, p_{12}=0, p_{13}=0 \Rightarrow p_d=1$	8	1	42.7	88.8
12	$p_3=1, p_{11}=0, p_{13}=0 \Rightarrow p_d=1$	18	1	38.8	94.7
13	$p_3=1, p_{11}=0, p_{12}=0 \Rightarrow p_d=1$	19	3	31.3	86.4
14	$p_3=1, p_{10}=0, p_{13}=0 \Rightarrow p_d=1$	15	3	42.7	83.3
15	$p_1=2, p_6=0, p_{16}=4 \Rightarrow p_d=1$	10	0	38.8	100
16	$p_1=2, p_3=1, p_{12}=0 \Rightarrow p_d=1$	9	0	37.3	100
17	$p_1=2, p_3=1, p_{10}=0 \Rightarrow p_d=1$	12	1	32.3	92.3
18	$p_{16}=3, p_{14}=0 \Rightarrow p_d=1$	17	2	42.7	89.4
19	$p_{15}=2, p_{14}=0 \Rightarrow p_d=1$	11	0	49.1	100
20	$p_{13}=0, p_{19}=3 \Rightarrow p_d=1$	13	0	31.3	100
21	$p_9=0, p_{14}=0 \Rightarrow p_d=1$	13	0	42.7	100
22	$p_6=0, p_{14}=0 \Rightarrow p_d=1$	16	1	42.7	94.1
23	$p_4=0, p_{14}=0 \Rightarrow p_d=1$	15	3	36.2	83.3
24	$p_{12}=1, p_{17}=2, p_{19}=1 \Rightarrow p_d=0$	9	0	32.3	100
25	$p_{12}=1, p_{13}=1, p_{18}=1 \Rightarrow p_d=0$	11	0	32.3	100
26	$p_9=1, p_{19}=1, p_{14}=1 \Rightarrow p_d=0$	8	1	38.8	88.8
27	$p_8=1, p_{12}=1, p_{13}=1 \Rightarrow p_d=0$	11	0	38.8	100
28	$p_7=0, p_{12}=1, p_{19}=1 \Rightarrow p_d=0$	15	2	49.5	88.2
29	$p_6=1, p_{16}=4, p_{19}=1 \Rightarrow p_d=0$	7	0	31.3	100
30	$p_6=1, p_7=0, p_{19}=1 \Rightarrow p_d=0$	13	1	31.3	92.8
31	$p_5=1, p_{16}=3, p_{19}=1 \Rightarrow p_d=0$	17	0	30.7	100
32	$p_1=3, p_{13}=1, p_{18}=1 \Rightarrow p_d=0$	9	0	38.4	100
33	$p_1=3, p_6=1, p_{19}=1 \Rightarrow p_d=0$	13	1	53.8	92.8
34	$p_3=1, p_1=3, p_{13}=1 \Rightarrow p_d=0$	12	2	38.4	85.7

**Figure 6:**
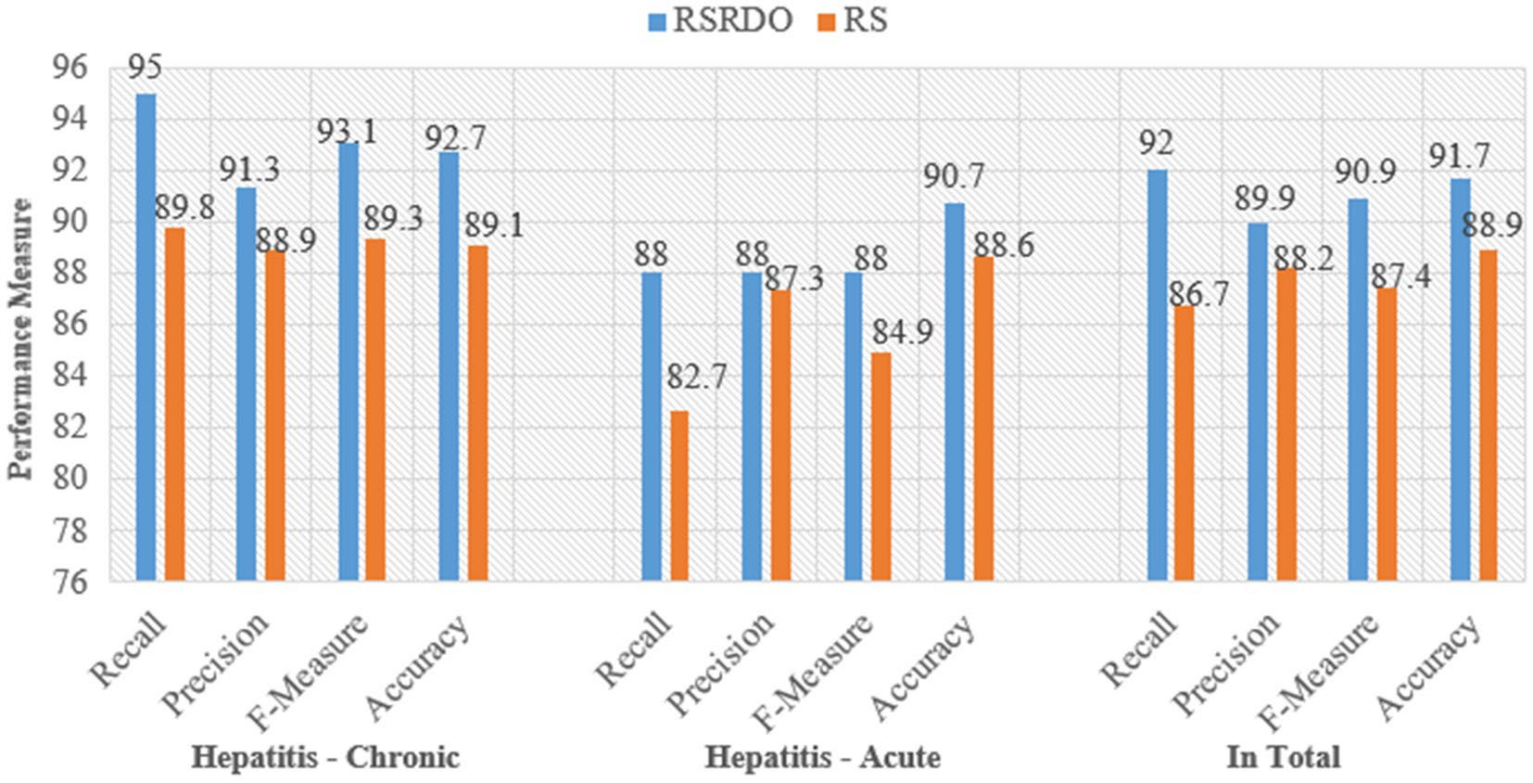
Measure of performance of RSRDO and RS.

Making use of 193 patients’ data from the testing dataset, the 34 decision rules are examined further in detail. All the decision rules have an accuracy of more than 65% and hence are selected. Moreover, the confusion matrix of the RS model, which takes into account all classes, is computed and shown in Table 11. Table 11 demonstrates that the RS model provides an accuracy of 88.9%. However, the RSRDO predictive accuracy is 91.7%. Because of this, the proposed RSRDO approach offers 2.8% more accuracy than the RS model. Figure 6 shows different measurements for the two models, RSRDO and RS to make them easier to understand.

**Table 11:** Performance measure of RS over hepatitis B disease.

Description	$t^n$	$t^p$	$f^n$	$f^p$	Recl. (%)	Precn. (%)	F-Measure (%)	Acc. (%)
$p_d=0$	84	88	10	11	89.8	88.8	89.3	89.1
$p_d=1$	109	62	13	9	82.6	87.3	84.9	88.6
Total	193	150	23	20	86.7	88.2	87.4	88.9

### Comparison analysis with DT

**Figure 7:**
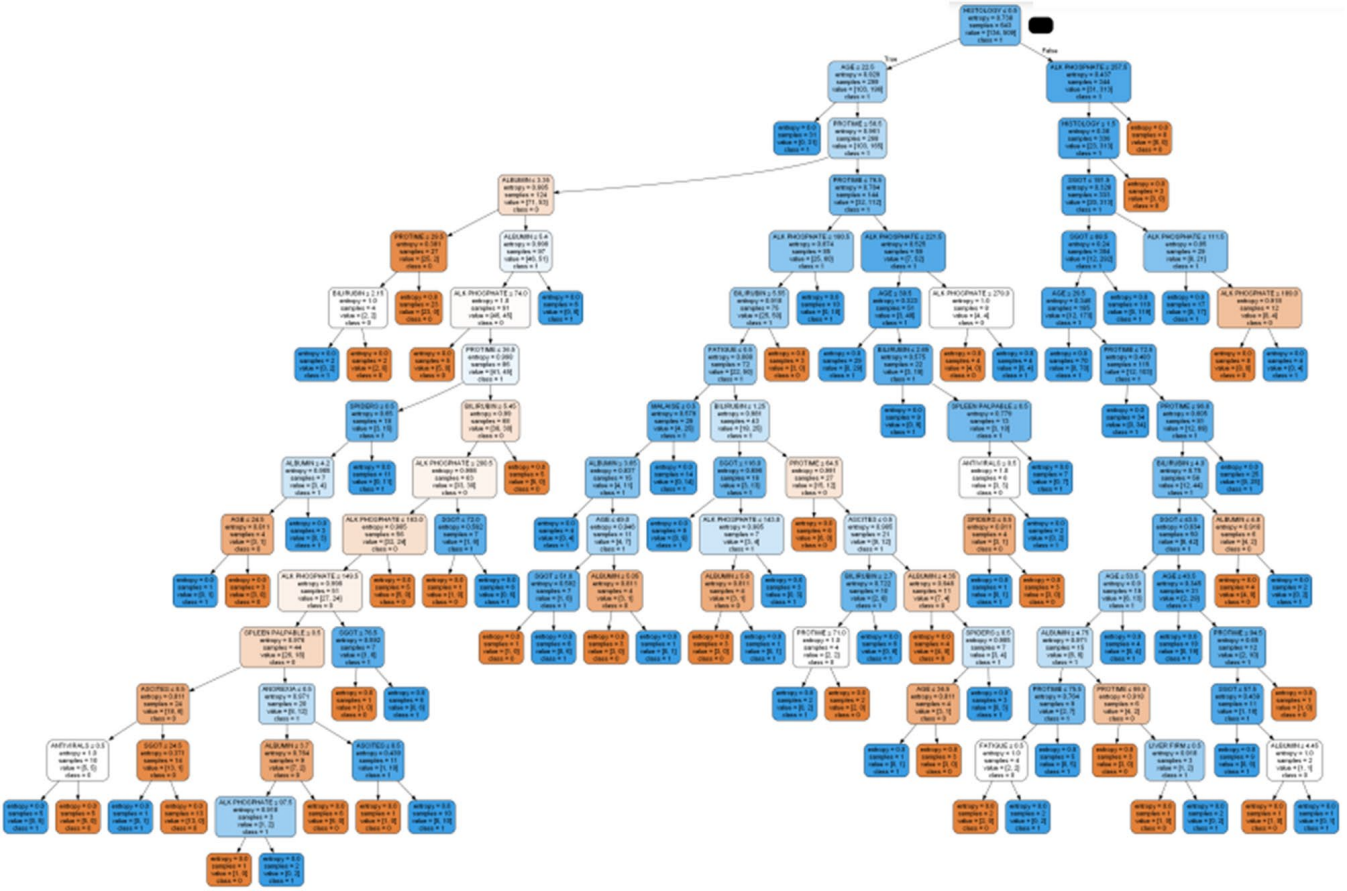
Decision tree approach decision rules.

The outcomes of the projected RSRDO approach and the Decision Tree (DT) approach have been compared in this Sect. ^38^. The DT approach is used to produce the decision rules while taking into account all 19 characteristics. DT algorithm is used to attain an accuracy of 82.9%. Figure 7 displays the decision rules that the DT procedure generated. It follows that the RSRDO model is 8.8% more accurate than the DT procedure. Similarly to this, the RS procedure is 6.0% more accurate than the DT procedure.

### Comparison analysis with RDO-DT approach

**Figure 8:**
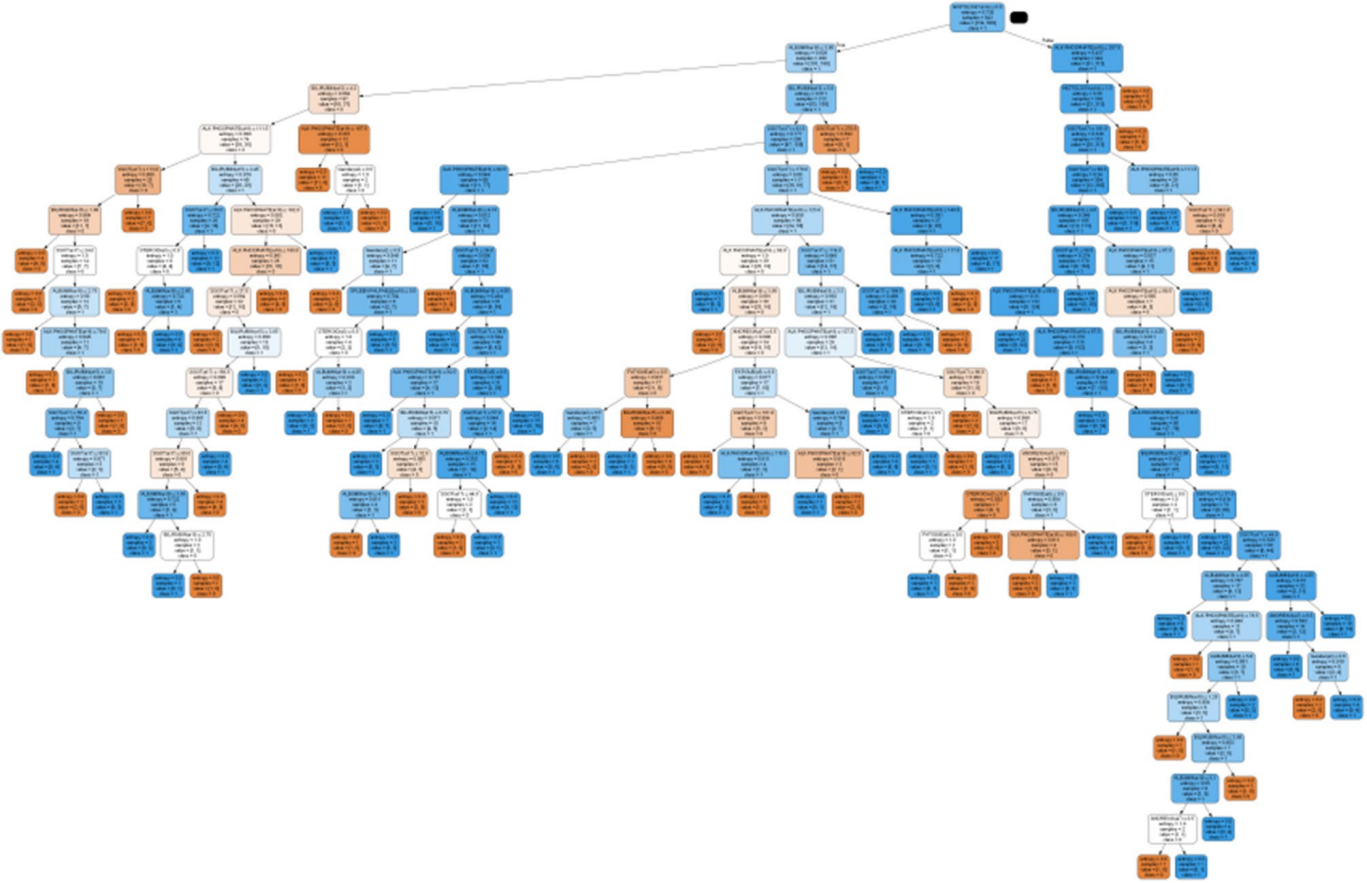
RDO—decision tree approach decision rules.

The outcomes of the projected RSRDO approach and the RDO-Decision Tree (RDODT) procedure have been compared in this section. DT approach is used to construct the decision rules while taking into account the chosen RDO approach characteristics. Using the RDODT technique, an accuracy of 88.6% is attained. The decision rules produced by the RDODT procedure are shown in Fig. 8. Consequently, it is evident that the DT approach has an accuracy of 5.8% lower than the RDODT procedure. In a similar vein, the RDODT model has 0.3% less accurate than the RS approach. However, the proposed RSRDO procedure is 3.1% more accurate than the RDODT procedure.

A comparative analysis of all models relating to recall, precision, f-measure, accuracy is presented in Table 12. From the analysis, it is clear that the proposed model RSRDO performs better across all the measures.

**Table 12:** Comparative performance measure of all models over hepatitis B disease.

Decision	Measure	RSRDO	RS	DT	RDODT
Hepatitis B Chronic	Recl. (%)	95.0	89.8	80.2	88.8
	Precn. (%)	91.3	88.8	80.2	88.8
	F-Measure (%)	93.1	89.3	80.2	88.8
	Acc. (%)	92.7	89.1	81.3	88.6
Hepatitis B Acute	Recl. (%)	88.0	82.6	80.6	83.8
	Precn. (%)	88.0	87.3	76.1	86.1
	F-Measure (%)	88.0	84.9	78.3	84.9
	Acc. (%)	90.7	88.6	84.5	88.6
Total	Recl. (%)	92.0	86.7	80.4	86.6
	Precn. (%)	89.9	88.2	78.4	87.6
	F-Measure (%)	90.9	87.4	79.4	87.1
	Acc. (%)	91.7	88.9	82.9	88.6

## Research contributions and limitations of the study

This section highlights the research contributions and limitations of this research work. In this study, the following contribution has been made.

1. To help doctors diagnose hepatitis B illnesses, a novel clinical information retrieval system that combines the RS and RD algorithms has been outlined.
2. Using a hepatitis B clinical information system, a novel feature selection approach that integrates RD optimization and the degree of dependency of the RS is given and examined.
3. The advocated RSRDO model’s experimental effectiveness is assessed over the assessment of hepatitis B.
4. In terms of accuracy, the suggested approach RSRDO is also contrasted with the RS, DT, and RDODT models. It is discovered that RSRDO outperforms other models despite having the fewest features in the decision system.
5. In comparison to the RS model, the suggested model RSRDO produces 55.9% more decision rules while achieving a high accuracy of 91.7%.

### Limitations of the experimental research

A clinical information retrieval system is developed by the integration of an RS and red deer algorithm in the suggested research study. Qualitative data are supported by the RS data analysis. Thus, with the assistance of specialized professionals, an attempt has been made to convert the information system’s continuous data values to subjective information. Without consulting specialized professionals, a fuzzy RS could be able to handle this more effectively. Similar to this, the RSRDO algorithm does not balance local and global search in feature selection. It is because, loudness of MRD in the search space promotes local search exploitation. Likewise, the manner in which combating between stags and commanders is taken into account in local searches to provide improved solutions. These two represent the main research work limitations that may be solved in further studies.

The proposed algorithm RSRDO is a meta-heuristic algorithm. On employing the data partition approach, the problem can be scaled to a larger datasets. Besides, RS supports parallel processing also. All meta-heuristics algorithms never provides optimal solution to all problems. In general the meta-heuristic algorithms are problem specific. So, the proposed RSRDO algorithm may not provide optimal solution to all the problems. It can be studied in future research. At present, it can be considered as a limitation.

## Conclusions

Each short while, more and more healthcare data are being gathered. Data often exhibits uncertainties, which is a frequent feature. It takes a lot of work to analyze such data and produce any useful information. In order to achieve this, this work introduces the RSRDO clinical information retrieval system, which combines RS and RDO for disease diagnosis. Over the hepatitis B diagnosis system, the integrated RSRDO clinical information retrieval system is being examined. Additionally, the classic RS, DT, and RDODT models are contrasted with the clinical information retrieval system RSRDO. The suggested RSRDO procedure outperforms all mentioned procedures with an accuracy of 91.7%. For the RS, DT, and RDODT procedures, the obtained accuracy is 88.9%, 82.9%, and 88.6%, respectively. The investigation shows that the RSRDO procedure is 2.8% more accurate than the RS procedure. Likewise, the RSRDO procedure is 8.8% more accurate than the DT procedure and 3.1% more accurate than the RDODT procedure. Furthermore, 55.9% more rules are generated by the suggested RSRDO model than by the conventional RS model.

This research has mainly several practical advantages. The projected clinical information retrieval system uses minimum number of features for the diagnosis of hepatitis B. The helps in reducing the cost of treatment for patients while diagnosis of hepatitis B. Besides, the clinical information system generates more number of rules with less number of features. These rules help the physician to look in deeper for the hepatitis B diagnosis. As a result, the disease can be detected at an early stage and it can save the life of a patient. The findings obtained through this research work, it is anticipated, will help doctors choose the best course of action.

In the information system, it is discovered that several features have continuous values. With the assistance of discipline experts, these continuous values of the features are classified. Therefore, improved accuracy could result from the hybridization of fuzzy RS and RDO. Furthermore, the decision rules acquired can be subjected to formal concept analysis to identify the primary influencing elements. It is intended as a future line of research.

## Data Availability

The supporting data set was gathered from the UCI repository^36^. Additionally, medical records from some primary health centers in West Bengal, India, are taken into account for the analysis. The UCI repository data is available in a publicly accessible repository, whereas the data collected from primary health centers in West Bengal, India will be made available on request due to restrictions such as privacy or ethics.
